# Enrichment of miR-17-5p enhances the protective effects of EPC-EXs on vascular and skeletal muscle injury in a diabetic hind limb ischemia model

**DOI:** 10.1186/s40659-023-00418-5

**Published:** 2023-04-01

**Authors:** Qunwen Pan, Xiaobing Xu, Wen He, Yan Wang, Zhi Xiang, Xiaojuan Jin, Qiong Tang, Ting Zhao, Xiaotang Ma

**Affiliations:** 1 Guangdong Key Laboratory of Age-Related Cardiac and Cerebral Diseases, Institute of Neurology, Affiliated Hospital of Guangdong Medical University, Zhanjiang, 524001 China; 2 Institute of Biochemistry and Molecular Biology, Guangdong Medical University, Zhanjiang, China; 3 Out-Patient Department, Affiliated Hospital of Guangdong Medical University, Zhanjiang, China

**Keywords:** Diabetic hind limb ischemia, Endothelial progenitor cells, Exosomes, miR-17-5p

## Abstract

**Background/aims:**

Diabetes mellitus (DM) is highly susceptible to diabetic hind limb ischemia (DHI). MicroRNA (MiR)-17-5p is downregulated in DM and plays a key role in vascular protection. Endothelial progenitor cell (EPC)-released exosomes (EPC-EXs) contribute to vascular protection and ischemic tissue repair by transferring their contained miRs to target cells. Here, we investigated whether miR-17-5p-enriched EPC-EXs (EPC-EXs^miR-17-5p^) had conspicuous effects on protecting vascular and skeletal muscle in DHI in vitro and in vivo.

**Methods:**

EPCs transfected with scrambled control or miR-17-5p mimics were used to generate EPC-EXs and EPC-EXs^miR-17-5p^. Db/db mice were subjected to hind limb ischemia. After the surgery, EPC-EXs and EPC-EXs^miR-17-5p^ were injected into the gastrocnemius muscle of the hind limb once every 7 days for 3 weeks. Blood flow, microvessel density, capillary angiogenesis, gastrocnemius muscle weight, structure integrity, and apoptosis in the hind limb were assessed. Vascular endothelial cells (ECs) and myoblast cells (C2C12 cells) were subjected to hypoxia plus high glucose (HG) and cocultured with EPC-EXs and EPC-EXs^miR-17-5p^. A bioinformatics assay was used to analyze the potential target gene of miR-17-5p, the levels of SPRED1, PI3K, phosphorylated Akt, cleaved caspase-9 and cleaved caspase-3 were measured, and a PI3K inhibitor (LY294002) was used for pathway analysis.

**Results:**

In the DHI mouse model, miR-17-5p was markedly decreased in hind limb vessels and muscle tissues, and infusion of EPC-EXs^miR-17-5p^ was more effective than EPC-EXs in increasing miR-17-5p levels, blood flow, microvessel density, and capillary angiogenesis, as well as in promoting muscle weight, force production and structural integrity while reducing apoptosis in gastrocnemius muscle. In Hypoxia plus HG-injured ECs and C2C12 cells, we found that EPC-EXs^miR-17-5p^ could deliver their carried miR-17-5p into target ECs and C2C12 cells and subsequently downregulate the target protein SPRED1 while increasing the levels of PI3K and phosphorylated Akt. EPC-EXs^miR-17-5p^ were more effective than EPC-EXs in decreasing apoptosis and necrosis while increasing viability, migration, and tube formation in Hypoxia plus HG-injured ECs and in decreasing apoptosis while increasing viability and myotube formation in C2C12 cells. These effects of EPC-EXs^miR-17-5p^ could be abolished by a PI3K inhibitor (LY294002).

**Conclusion:**

Our results suggest that miR-17-5p promotes the beneficial effects of EPC-EXs on DHI by protecting vascular ECs and muscle cell functions.

**Supplementary Information:**

The online version contains supplementary material available at 10.1186/s40659-023-00418-5.

## Introduction

Diabetes mellitus (DM) is associated with vascular damage, ischemic injury and abnormal tissue repair, resulting in hind limb ischemia, myocardial infarction, ischemic stroke and other complications [1]. Diabetic hind limb ischemia (DHI) is one of the most common complications related to DM, accounting for 30% of DM complications. In severe cases, diabetic foot can result in amputation or even death [2], and effective therapeutic strategies are still lacking. Evidence has demonstrated that the scarcity of microvessels, obstruction of vascular formation, and impairment of muscle tissue in hind limb ischemia are important factors contributing to DHI. Endothelial cells (ECs) play important roles in vascular integrity and angiogenesis [3]. It is well known that myoblast-induced myogenic differentiation and myotube formation are key steps in muscle regeneration [4]. Thus, a therapeutic approach targeting both EC and myoblast protection should be important for ameliorating DHI.

Endothelial progenitor cells (EPCs) are a subtype of progenitor cells that can differentiate into mature ECs, which participate in the generation of neovascularization and the repair of injured tissues through the secretion of growth cytokines and angiogenic factors [5, 6]. We and others have shown that EPCs exert therapeutic effects on various ischemia animal models, such as ischemic stroke, hind limb ischemia, and myocardial infarction [5, 7]. However, the survival rate and therapeutic efficacy of EPCs can be impaired by the diabetic environment [8]. Of note, increasing evidence has demonstrated that the therapeutic efficacies of stem cells are highly dependent on their released exosomes (EXs) [9, 10]). EXs are nanosized extracellular vesicles that can affect recipient cell functions by transferring their cargos, including proteins and microRNAs (miRs) [9]. Human CD34 stem cell EXs have been shown to improve ischemic hind limb perfusion and capillary density by transferring miR-126-3p [11]. Adipose stem cell-released EXs can promote vascularization and cutaneous wound healing after hind limb ischemia in diabetic rats [10]. Compared to stem cells, stem cell-derived EXs capably pass through the barrier structure, with stable function under the pathological environment [9] and less risk of oncogenesis and rejection reactions in the body [12]. Recently, numerous studies have demonstrated that EXs derived from EPCs (EPC-EXs) can improve hypoxia/reoxygenation-injured EC functions and promote angiogenesis in the peri-infarct area of ischemic stroke mice [9, 12]. In addition, EPC-EXs have been shown to reduce myocardial infarction by promoting cardiac fibroblast proliferation and angiogenesis [13]. However, whether EPC-EXs can protect against HG- and hypoxia-induced EC and myoblast dysfunction and exert therapeutic effects on DHI is unknown.

MiRs are functional cargos in EXs and are implicated in the occurrence and development of DM [14]. Recent evidence has demonstrated that miR-126 can promote the therapeutic effects of EPC-EXs in diabetic ischemic stroke [9]. Thus, the use of miR-engineered EPC-EXs has the potential to be an effective new method for treating DM and diabetes complications. MiR-17-5p is downregulated in the peripheral blood of diabetic patients [15] and has been shown to contribute to the anti-inflammatory and anti-apoptotic effects of baicalein on human trophoblasts subjected to high glucose [16]. In hypoxia/reoxygenation (H/R)-injured ECs, miR-17-5p has been shown to increase cell viability and inhibit cell apoptosis [17]. Additionally, there is evidence showing that miR-17-5p can promote C2C12 myoblast differentiation [18]. MiR-17-5p has been implicated in myocardial ischemia‒reperfusion injury [19, 20] and can also improve the vascular repair of aneurysms by promoting the endothelialization of EPCs by regulating the PTEN/PI3K/Akt/VEGFA pathway [21]. A recent study demonstrated that extracellular vesicles from mesenchymal stem cells promoted diabetic wound healing through miR-17-5p-mediated enhancement of angiogenesis [22]. Additionally, Li W et al. found that EXs derived from mesenchymal stem cells can reduce oxidative injury and retinal cell apoptosis in diabetic retinopathy mice by transferring their contained miR-17 [23]. However, the effects and underlying mechanism of EPC-EXs combined with miR-17-5p in hind limb vascular and muscle damage after diabetic hind limb ischemia injury are unknown. Our preliminary study showed that miR-17-5p was downregulated in hind limb vessels and muscle tissues. Moreover, we observed that SPRED1 was a downstream target of miR-17-5p, and SPRED1-PI3K/Akt has been found to be involved in regulating multiple cell functions, including EC proliferation, migration and tube formation abilities [24] and ischemic muscle cell repair [11]. Based on these findings, we hypothesize that enrichment of miR-17-5p may increase the beneficial effects of EPC-EXs on protecting ECs and myocyte cells from HG plus hypoxia-induced injury by inhibiting SPRED1 expression and activating the PI3K/Akt signaling pathway.

In this study, we investigated the beneficial effects of miR-17-5p-enriched EPC-EXs on blood vessel and muscle damage in a DHI mouse model and further evaluated their roles in regulating EC and C2C12 myoblast functions under HG plus hypoxia conditions. The underlying mechanisms were explored by analyzing the SPRED1-PI3K/Akt signaling pathway.

## Materials and methods

### Cell culture

Human umbilical vein endothelial cells (ECs) were obtained from Zhong Qiao Xin Zhou Biotechnology Co., Ltd. (Shanghai, China). The cells were cultured in endothelial cell medium supplemented with 10% fetal bovine serum (FBS, Gibco, USA), 100 U/ml penicillin and 100 U/ml streptomycin in a 37 °C incubator with a humidified atmosphere of 5% CO_2_/95% air. C2C12 murine myoblasts (Procell, China) were cultured in flushing DMEM supplemented with 10% FBS and 1% penicillin‒streptomycin at 37 °C with 5% CO_2_. After C2C12 cells grew to confluence, the medium was replaced with differentiation DMEM supplemented with 2% horse serum (Procell) and 1% penicillin‒streptomycin.

Endothelial progenitor cells (EPCs) were obtained from the bone marrow of C57BL/6 mice and characterized as we previously described [25]. In brief, bone marrow was flushed out from tibias and femurs, and bone marrow mononuclear cells were isolated by using the density gradient centrifuge method. Bone marrow mononuclear cells isolated from mice were plated on fibronectin-coated 24-well plates and then grown in endothelial cell basal medium-2 supplemented with 5% fetal bovine serum containing EPC growth cytokine cocktail (Lonza, USA). After three days in culture, nonadherent cells were removed by washing with PBS. The culture medium was then changed every 2 days. After 2 weeks, adherent cells were incubated in 2.4 μg/ml Di-acLDL solution for 1 h, incubated in 10 μg/ml Bs-Lectin solution for 1 h, and observed under a fluorescence microscope. The red and green double-stained cells were identified as EPCs.

### EPC transfection

The lentivirus carrying murine miR-17-5p (Lv-miR-17-5p) or scrambled control (Lv-SC) was purchased from Genepharma (Shanghai, China). EPCs were transfected with Lv-miR-17-5p or Lv-SC to obtain miR-17-5p-overexpressing EPCs (EPC^miR-17-5p^) and controls (EPC^SC^) based on the manufacturer’s instructions. After lentiviral infection, puromycin (1 μg/ml) was added to select stably transduced EPCs. The positive cells were observed under a fluorescence microscope, and the transduction efficiency (the expression of miR-17-5p in EPCs) was confirmed by qRT‒PCR.

### Preparation and identification of EXs

EXs were isolated from serum-free culture medium of EPCs as we previously described [26]. Briefly, cells (EPC^SC^, EPC^miR-17-5p^) were placed in 100 mm plates for 48 h. Then, the medium was centrifuged at 2000×*g* for 20 min to remove cells and debris. The collected supernatants were ultracentrifuged at 20,000×*g* for 90 min and then at 100,000×*g* for 90 min to pellet EXs (EPC-EXs, EPC-EXs^miR-17-5p^). The pelleted EXs were resuspended in filtered PBS and used for nanoparticle tract analysis (NTA), transmission electron microscopy (TEM), and EX-specific marker (CD63 and TSG101) analysis.

### Nanoparticle tracking analysis

The concentration and size distribution of EXs were measured by nanoparticle tracking analysis (NTA) as we previously described [7]. In brief, suspended EXs were diluted in 1 ml PBS and applied to NanoSight (NS300) to automatically measure the average diameter and concentration.

### Transmission electron microscopy

For electron microscopy, 10 μl suspended EXs were pipetted onto carbon-coated copper grids. After the sample was dry, micrographs were taken with a calibrated magnification of 100,000-fold by transmission electron microscopy (TEM).

### HG and hypoxia injury model of ECs and C2C12 cells

ECs and C2C12 cells were subjected to high glucose (HG) plus hypoxia treatment as described in a previous study with minor modifications [27]. After coculture with EXs (EPC-EXs, EPC-EXs^miR-17-5p^), ECs and C2C12 cells were incubated with HG (25 mM) for 24 h and then maintained under hypoxic conditions (1% O_2_, 5% CO_2_, 94% N_2_) for 6 h. The cells incubated with PBS and then cultured under hypoxic plus HG conditions were set as the vehicle group. Meanwhile, cells cultured under normoxic conditions were used as controls. Cells were harvested for RT‒qPCR analysis and assays of viability, apoptosis, necrosis, cell diameter of myotubes (for C2C12 cells), and tube formation (for ECs).

### Coculture assay of EPC-EXs with target cells

EXs were labeled with PKH26 (2 µM, a red fluorescence cell membrane dye) according to the manufacturer’s protocol. The PKH26-labeled EPC-EXs were cocultured with ECs or C2C12 cells for 24 h. Then, the cells were washed with PBS and incubated with fluorescein isothiocyanate (FITC)-conjugated anti-beta actin (Abcam, USA). The incorporation of EXs into ECs (or C2C12 cells) was examined under a fluorescence microscope (Leica, Germany).

### Real-time quantitative PCR

Total miR from EPCs, EPC-EXs, hind limb vessels and muscle tissue was extracted using a miRNeasy Mini Kit (QIAGEN) according to the manufacturer’s instructions. miR-17-5p cDNA was synthesized using a Hairpin-it™ miR RT‒PCR Quantitation Kit (GenePharma, Shanghai, China) at 25 °C for 30 min, 42 °C for 30 min, and 85 °C for 5 min. Real-time PCR was conducted on an RT‒PCR system (Bio-Rad). The parameters were 95 °C for 3 min; 40 cycles performed at 95 °C for 12 s and 60 °C for 40 s. PCR primers were as follows: (miR-17-5p: forward, 5′-TGCGCCAAAGTGCTTACAGTGCA-3′ and reverse, 5′-CCAGTGCAGGGTCCGAGGTATT-3′, GAPDH: forward: 5′-GGAGCGAGATCCCTCCAAAAT-3′, reverse: 5′-GGCTGTTGTCATACTTCT CATGG-3′.) The level of miR-17-5p was normalized to GAPDH. The relative quantification of gene expression was determined using the comparative CT method (2^−△△Ct^).

### MiR-17-5p targeted gene prediction

Bioinformatics prediction of target genes and binding sites of miR-17-5p was performed using the TargetScan database (http://www.targetscan.org/vert_72/), miRbase (https://www.mirbase.org/) and miRanda (http://www.microrna.org/). SPRED1 was found to be one of the overlapping targets for miR-17-5p.

### Cell viability, necrosis and apoptosis assays

The viability and apoptosis of ECs and C2C12 cells were detected as described in our previous study [26]. In brief, cell viability was detected by a CCK-8 assay kit (Beyotime, China) based on the manufacturer’s instructions. Cell apoptosis was measured by using an Annexin V-PE/7-AAD apoptosis detection kit (BD bioscience, USA) followed by flow cytometric analysis. EC necrosis was measured by using an Apoptosis and Necrosis Detection Kit (Beyotime, China) based on the manufacturer’s instructions, followed by immunofluorescence analysis. After coculture with EXs, ECs were incubated with 1 ml of YPI/PI working solution at 37 °C for 20 min and then observed under a fluorescence microscope. The cells that were double positive for YO-PRO-1 and PI staining were considered necrotic ECs.

### EC tube formation analysis

The tube formation ability of ECs was detected as reported in our previous study [26]. In brief, ECs were placed onto an EC matrix and cultured with endothelial cell growth medium for 12 h. The formed tubes were observed under an inverted light microscope, and the average number of tubes per field was counted.

### C2C12 myotube size analysis

The myotube size of C2C12 cells was measured as described in a previous study [4]. Briefly, C2C12 myoblasts were cultured in differentiation medium supplemented with 2% horse serum for 3 d, and then the myotubes were stained with rabbit anti-MyHc antibody at 4 °C overnight, followed by incubation with Alexa Fluor-conjugated goat anti-rabbit secondary antibody for 1 h at room temperature. Myotube sizes were photographed under a confocal microscope (Olympus, Japan). The diameter of each myotube was detected using NIH ImageJ software.

### Animals

Adult male db/db type II mice (8–12 weeks old) purchased from the Animal Experiment Center of Guangdong Province (Guangzhou, China) were subjected to hind limb ischemia surgery. The mice were housed in a pathogen-free environment, and surgeries were performed under 2.5% isoflurane anesthesia. All experimental procedures were approved by the laboratory animal care and use committees at Guangdong Medical University.

### Administration of EXs for hind limb ischemia mouse model

Db/db diabetic mice were used to induce a hind limb ischemia (DHI) model as described in a previous study [28]. Briefly, after anesthesia, the femoral artery and the upper end of the artery bifurcation near the knee were separated and ligated. The main branch of the femoral artery between the double nodes was blocked to establish the DHI model. Posthind limb ischemia, mice were intramuscularly injected with PBS (vehicle), EPC-EXs, EPC-EXs^miR-17-5p^ (1 × 10^11^ EXs/100 μl) once every 7 d. At 21 d after DHI, the mice were assessed using various measurements, including EPC-EX colocalization, hind limb blood flow (HBF), immunofluorescence and histology analysis. The fasting blood glucose level was measured using an ACCU-CHEK Performa glucometer (Roche, Basel, Switzerland). All measurements were performed by an investigator who was blinded to the grouping information.

### Measurements of HBF and microvascular density

The HBF of mice was assessed according to a previous study described with modifications [29]. Diabetic mice were anesthetized and placed on a stereotaxic apparatus. PeriCam PSI system (Perimed, Sweden) scanning was performed on the hind limb for approximately 1 min. The relative HBF was calculated using the following formula: HBF of ischemic hind limb/HBF of normal hind limb × 100%.

### Muscle force analysis

The muscle force of mice was evaluated according to a previous study [30]. Briefly, mice were initially anesthetized using 4% isoflurane and maintained by air inhalation through a facemask continuously supplied with 1.5% isoflurane. Electrical stimuli were delivered through two electrodes located below the knee and the Achilles tendon. The right foot was positioned to allow for the detection of hind limb muscle force. Maximal isometric tetanic tension was measured at different stimulating frequencies (10, 20, 30, 50, and 100 Hz, 1 s train duration), with 1 min between each stimulation train.

### Immunofluorescence analysis

EPC-EX colocalization with microvascular endothelial cells (CD31) and hind limb muscle cells (Myod1) was measured as described in a previous study with modifications [9]. PKH26-labeled EPC-EXs were intramuscularly injected into mice. After 2 d of administration, the hind limb tissue was isolated and cryosectioned (20 μm). Sections were fixed, permeabilized, and incubated with anti-CD31, anti-Ki67 or anti-Myod1 at 4 °C overnight, followed by incubation with Alexa Fluor- or FITC-conjugated anti-mouse/rabbit secondary antibodies for 1 h at room temperature. The colocalization of EPC-EXs with microvascular endothelial cells or hind limb muscle cells was observed under a confocal microscope (Olympus, Japan).

The microvascular density (MVD) was detected as described in a previous study by immunofluorescence staining [29]. In brief, the tibialis anterior muscles from normal diabetic or DHI mice were cryosectioned and incubated with CD31 antibody (1:50, Abcam) overnight. Then, the muscle sections were incubated with Alexa Fluor-conjugated secondary antibody (1:500, Abcam) for 1 h. DAPI dye was used to stain the cell nuclei. Images were observed under a confocal microscope (Olympus, Japan) to determine the MVD.

For angiogenesis, BrdU (IP, 65 μg/g per day) was administered by intraperitoneal injection after EPC-EX infusion for 7 contiguous days. Hind limb muscle sections were incubated with BrdU (1:50; Abcam) and anti-CD31 overnight at 4 °C and then incubated with FITC- or Alexia-conjugated secondary antibodies for 1 h. Angiogenesis in hind limb ischemic tissue was determined as BrdU + CD31 + cells.

For hind limb vascular EC viability analysis, hind limb muscle sections were incubated with Ki67 (1:100; Abcam) and anti-CD31 antibodies overnight at 4 °C and then incubated with FITC- or Alexia-conjugated secondary antibodies for 1 h. Hind limb vascular EC viability in hind limb ischemic tissue was determined as Ki67 + CD31 + cells.

Microvascular endothelial cell apoptosis was analyzed by a TUNEL assay kit (Beyotime) based on the manufacturer’s protocols. The hind limb muscle tissue sections were incubated with anti-CD31 for microvascular endothelial cells, followed by incubation with Alexia-conjugated secondary antibody. The slices were then incubated with TUNEL working solution for 60 min at room temperature. DAPI was used to stain nuclei. The labeled TUNEL + CD31 + cells were considered apoptotic microvascular endothelial cells in hind limb tissue.

### Histological analysis

At Day 21, mice were anesthetized, and hind limb muscles were collected and examined for gross abnormalities, weighed and stored in formalin for histological analysis. The structural integrity of the ischemic muscles was detected using Masson’s trichrome staining based on the manufacturer’s instructions and a previous study [31]. Images were acquired by a light microscope and quantitated using ImageJ software.

### Western blot analysis

ECs or C2C12 cells were homogenized in lysis buffer containing proteinase and phosphate kinase inhibitors (Sigma, USA). Proteins (30 μg) were mixed with loading buffer, electrophoresed through SDS‒PAGE gels and transferred to polyvinylidene difluoride membranes (PVDF, Millipore Corporation, USA). After blocking nonspecific antigens in 5% nonfat milk, the membranes were incubated with primary antibodies against β-actin (1:4000; Sigma), SPRED1 and PI3K (1:1000, Abcam), Akt and phospho-Akt (1:1000, CST, USA), cleaved caspase-3 and cleaved caspase-9 (1:500, Abcam), and TSG101 and CD63 (1:400, Abcam). Afterward, the membranes were washed and incubated with horseradish peroxidase-conjugated anti-rabbit or anti-mouse IgG (1:40,000; EarthOx) for 1 h at room temperature. The bands were visualized using an ECL kit (Amersham, Sweden).

### Statistical analysis

All data are expressed as the mean ± SEM (standard error of the mean). Comparisons for two groups were analyzed by independent T tests. GraphPad Prism 7 software was used to analyze the data. Multiple comparisons among groups were analyzed by the One-/-two-way ANOVA, followed by Tukey’s post hoc test (SPSS 25, USA). For all measurements, a p < 0.05 was considered statistically significant.

## Results

### MiR-17-5p transfection increased the levels of miR-17-5p in EPCs and EPC-EXs

**Fig. 1:**
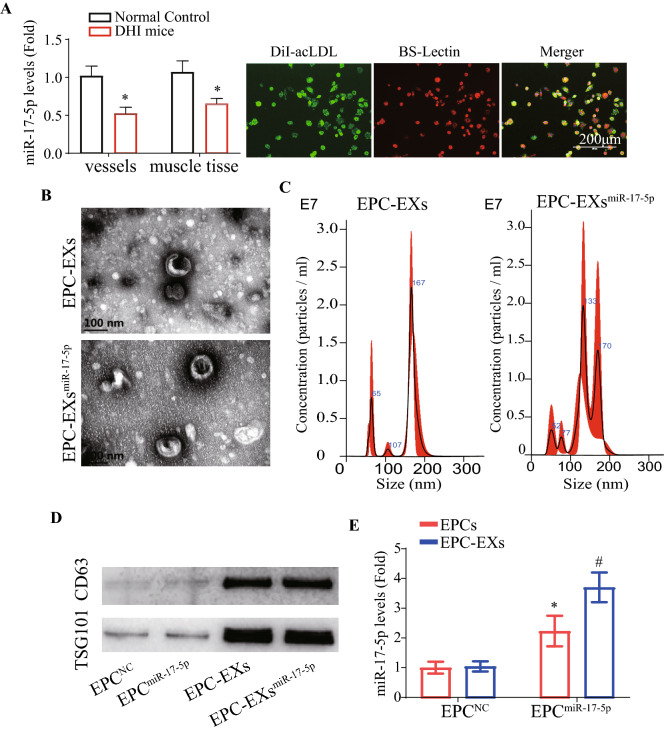
miR-17-5p enriched EPC-EXs preparation and characterization. **A** The miR-17-5p level in hind limb vessels and muscle tissues. **B** Representative images showing cultured EPCs by double staining analysis. Red: Di-LDL; Green: Bs-Lectin. Scale bar: 200 μm. **C** The morphology of EPC-EXs was detected by TEM. Scale bar, 200 μm. **D** The level and size of EPC-EXs was detected by NTA. **E** EXs specific markers CD63 and TSG101 were detected by western blotting. **F** The miR-17-5p level in EPCs and EPC-EXs (**A**, **E** Independent t test. Data represent the mean ± SEM, n = 3 mice per group. ^*^p < 0.05)

As shown in Fig. 1, we found that miR-17-5p was significantly decreased in the hind limb vessels and muscle tissues of DHI mice (vs. Normal Control, Fig. 1A, p < 0.05). Primary EPCs were isolated from bone marrow and defined as Dil-acLDL- and Bs-lectin-positive cells (Fig. 1B). The obtained EPC-EXs and EPC-EXs^miR-17-5p^ were characterized by NTA, TEM and western blotting. The results showed that they were similar in size (approximately 100 nm) and concentration (Fig. 1C, D, p > 0.05) and positively expressed the EX-specific markers CD63 and TSG101 (Fig. 1E). As shown in Fig. 1F, miR-17-5p transfection significantly increased the levels of miR-17-5p in EPCs and EPC-EXs (p < 0.05). These data demonstrated that miR-17-5p transfection induced the enrichment of miR-17-5p in EPCs and their secreted EXs.

### EPC-EXs^miR-17-5p^ were more effective than EPC-EXs in improving blood perfusion and angiogenesis in ischemic hind limbs of DHI mice

**Fig. 2:**
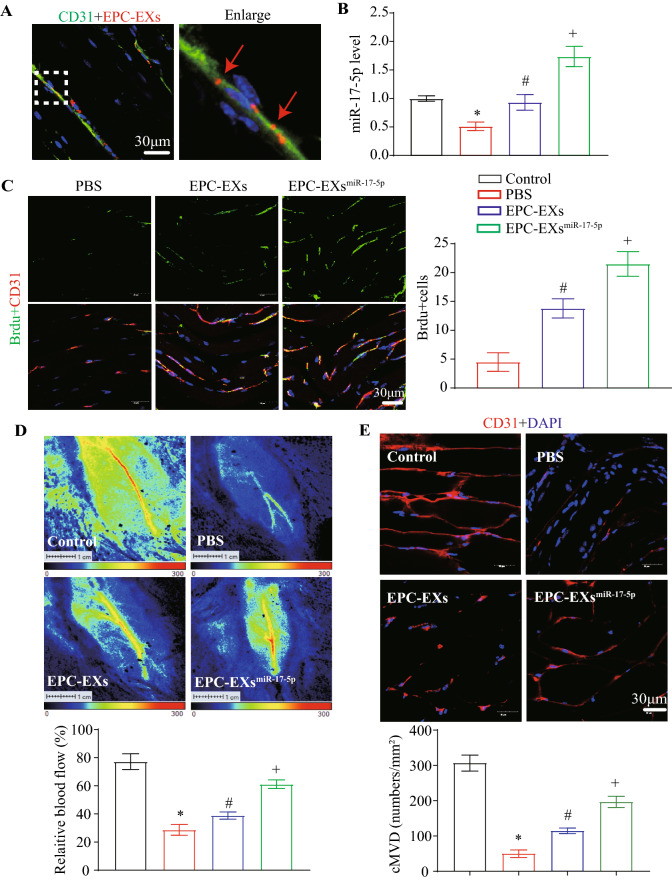
EPC-EXs^miR-17-5p^ infusion increases miR-17-5p levels, angiogenesis, blood perfusion, and MVD in hind limb of DHI mice. **A** Images showing PKH 26 labeled EPC-EXs (red) merged into vascular ECs (CD31, green). Scale bar, 30 μm. **B** The levels of miR-17-5p in hind limb vessels after EPC-EXs and EPC-EXs^miR-17-5p^ administration. **C** Representative images and summary data of angiogenesis in hind limb tissue of DHI mice. **D** Representative images and summary data of blood perfusion in hind limb tissue of DHI mice. **E** Representative images and summary data of MVD (CD31, red; DAPI, blue) in hind limb tissue of DHI mice. ^*^p < 0.05 compared with the Sham group; ^#^p < 0.05 compared with PBS group; ^+^p < 0.05 compared with EPC-EXs group (**B**, **D**, **E** Two-way ANOVA, followed by Tukey’s post hoc test; C One-way ANOVA, followed by Tukey’s post hoc test. Data represent the mean ± SEM, n = 8 mice per group)

As shown in Fig. 2A, fluorescent PKH26-labeled EPC-EXs^miR-17-5p^ were observed in microvascular ECs in the hind limbs of DHI mice at 48 h after EX injection. By RT‒qPCR, we found that administration of EPC-EXs^miR-17-5p^ markedly increased miR-17-5p expression in ischemic hind limb vessels (vs. EPC-EXs or vehicle, Fig. 2B, p < 0.05), suggesting that EPC-EXs^miR-17-5p^ could transfer their contained miR-17-5p to hind limb microvascular ECs. Twenty-one days after EX administration, we found that EPC-EXs were able to increase hind limb blood flow, MVD, and angiogenesis, which were compromised by hind limb ischemia treatment (vs. vehicle, Fig. 2C–E, p < 0.05), and EPC-EXs^miR-17-5p^ were more effective (vs. EPC-EXs, Fig. 2C–E, p < 0.05). In addition, we found that EPC-EXs were able to increase the proliferation of hind limb vascular ECs, which was compromised by hind limb ischemia treatment (vs. vehicle, Additional file 1: Fig. S1A, p < 0.05), and EPC-EXs^miR-17-5p^ were more effective (vs. EPC-EXs, Additional file 1: Fig. S1A, p < 0.05). In addition, we added an experiment to evaluate glycemia in mice with diabetic hind limb ischemia. We found that glycemia did not change significantly after EPC-EXs or EPC-EXs^miR-17-5p^ administration. Taken together, these results suggested that EPC-EXs^miR-17-5p^ were more effective than EPC-EXs in promoting hind limb blood perfusion and angiogenesis in ischemic hind limb tissue by transferring their contained miR-17-5p.

### EPC-EXs^miR-17-5p^ were more effective than EPC-EXs in protecting muscles in the ischemic hind limbs of DHI mice

**Fig. 3:**
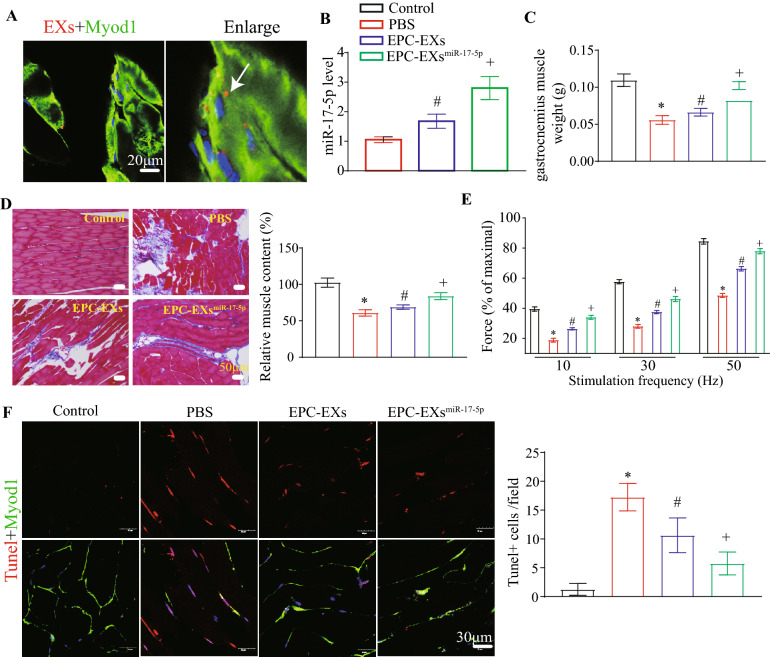
EPC-EXs^miR-17-5p^ infusion increases miR-17-5p levels, gastrocnemius muscle weight and structure integrity, while reduce apoptosis in hind limb of DHI mice. **A** Images showing PKH 26 labeled EPC-EXs (red) merged into muscle cell (Myod1, green). Scale bar, 20 μm. **B** The levels of miR-17-5p in hind limb muscle tissue after EPC-EXs and EPC-EXs^miR-17-5p^ administration. **C** Summary data of gastrocnemius muscle weight in DHI mice. **D** The structural integrity of ischemic muscles was measured by Masson’s staining. **E** Summary data of force generation capacity of muscle in DHI mice. **F** Representative images and summary data of cell apoptosis in hind limb of DHI mice. ^*^p < 0.05 compared with the Sham group; ^#^p < 0.05 compared with PBS group; ^+^p < 0.05 compared with EPC-EXs group (**B**–**F** Two-way ANOVA, followed by Tukey’s post hoc test. Data represent the mean ± SEM, n = 8 mice per group)

Fluorescent PKH26-labeled EPC-EXs^miR-17-5p^ were incorporated into muscle cells according to immunofluorescence analysis (Fig. 3A), and miR-17-5p expression was significantly increased in the hind limb muscle tissue of DHI mice (vs. EPC-EXs or vehicle, Fig. 3B, p < 0.05), suggesting that EPC-EXs^miR-17-5p^ could transfer their contained miR-17-5p to hind limb muscle cells. Morphometric analysis showed that the weight of the gastrocnemius muscle of the hind limb in DHI mice could be increased by EPC-EX treatment at 21 d after administration, which was compromised by hind limb ischemia treatment (vs. vehicle, Fig. 3C, p < 0.05), and EPC-EXs^miR-17-5p^ displayed better efficacy (vs. EPC-EXs, Fig. 3C, p < 0.05). By Masson’s trichrome staining assay, we observed that the structural integrity of the ischemic hind limb muscles could be improved by EPC-EX treatment, which was compromised by hind limb ischemia treatment (vs. vehicle, Fig. 3D, p < 0.05), and EPC-EXs^miR-17-5p^ were more effective (vs. EPC-EXs, Fig. 3D, p < 0.05). Additionally, we performed experiments to detect the force generation capacity of muscle in diabetic hind limb ischemia mice. We found that the force of the ischemic hind limb muscles, which was compromised by hind limb ischemia treatment, could be significantly improved by EPC-EX treatment (vs. vehicle, Fig. 3E, p < 0.05), and EPC-EXs^miR-17-5p^ were more effective (vs. EPC-EXs, Fig. 3E, p < 0.05). These results indicate that miR-17-5p-enriched EPC-EXs play an important role in the gain of muscle function. The immunofluorescence staining of TUNEL and DAPI further showed that EPC-EXs could reduce cell apoptosis of hind limb muscles in DHI mice (vs. vehicle, Fig. 3F, p < 0.05), and EPC-EXs^miR-17-5p^ displayed better efficacy (vs. EPC-EXs, Fig. 3F, p < 0.05).

Taken together, these results suggested that EPC-EXs^miR-17-5p^ were more effective than EPC-EXs in protecting muscles in ischemic hind limbs by transferring their contained miR-17-5p.

### EPC-EXs.^miR-17-5p^ were more effective than EPC-EXs in activating the PI3K/Akt signaling pathway in Hypoxia plus HG-treated ECs and C2C12 cells by targeting SPRED1

**Fig. 4:**
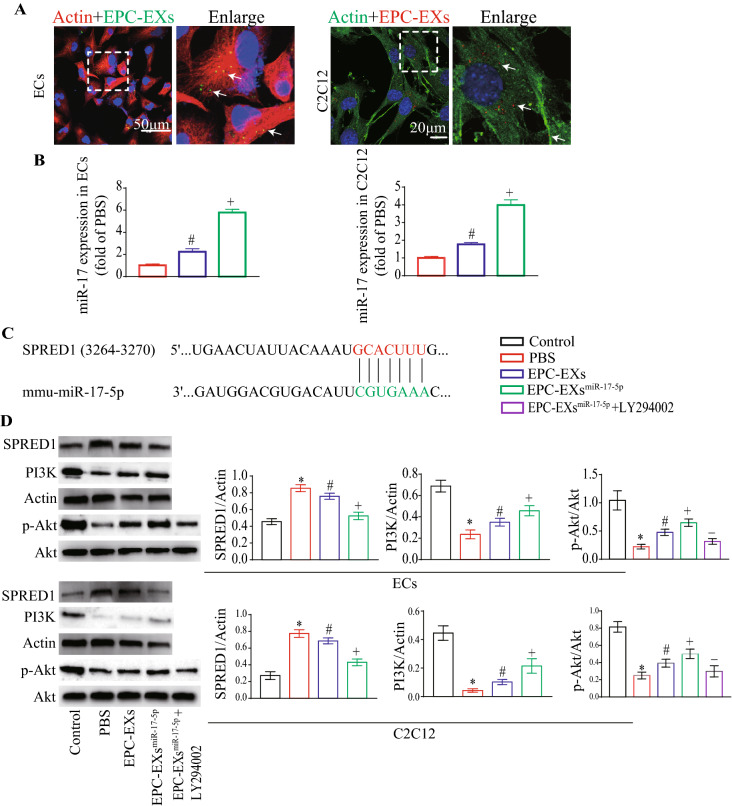
EPC-EXs.^miR-17-5p^ induced inhibition of SPRED1 and activation of the PI3K/Akt signaling pathway in Hypoxia plus HG-injured ECs or C2C12. **A** Immunofluorescence images of PKH26 labeled EPC-EXs merged into ECs and C2C12. **B** The levels of miR-17-5p in ECs and C2C12 after incubation with different EPC-EXs. **C** SPRED1 is a potential target of miR-17-5p. **D** The levels of SPRED1, PI3K, and p-Akt/Akt in ECs and C2C12 after incubation with different EPC-EXs (**B** One-way ANOVA, followed by Tukey’s post hoc test; **D** Two-way ANOVA, followed by Tukey’s post hoc test. Data represent the mean ± SEM. n = 3/group)

After coculture, PKH26-labeled EPC-EXs were observed in the cytoplasm of ECs and C2C12 cells (Fig. 4A), indicating that EPC-EXs can merge into target cells. Using RT‒qPCR, we found that EPC-EXs^miR-17-5p^ incubation markedly increased miR-17-5p expression in recipient ECs and C2C12 cells (vs. EPC-EXs, Fig. 4B, p < 0.05). These results suggest that miR-17-5p can be transferred from EPCs to ECs and C2C12 cells by EPC-EXs^miR-17-5p^.

By bioinformatics prediction analysis, we found that SPRED1 was a target of miR-17-5p (Fig. 4C). Consistent with our expectation, the expression levels of SPRED1 in hypoxia plus HG-treated ECs and C2C12 cells were markedly decreased after EPC-EX incubation (vs. vehicle, Fig. 4D, p < 0.05) and were further reduced by EPC-EX^miR-17-5p^ incubation (vs. EPC-EXs, Fig. 4D, p < 0.05). The downstream PI3K/Akt pathway proteins of miR-17-5p were further examined by western blotting. We found that EPC-EXs^miR-17-5p^ were more effective in increasing PI3K expression and the phosphorylation of Akt compared with the EPC-EXs-treated group (vs. EPC-EXs, Fig. 4D, p < 0.05), and application of a PI3K inhibitor (LY294002) partially abolished the effects of EPC-EXs^miR-17-5p^ on increasing the phosphorylation of Akt in Hypoxia plus HG-treated ECs and C2C12 cells (vs. EPC-EXs^miR-17-5p^, Fig. 4D, p < 0.05). These results indicate that miR-17-5p enrichment enhances the effects of EPC-EXs on activating the PI3K/Akt signaling pathway by targeting SPRED1 in Hypoxia plus HG-treated ECs and C2C12 cells.

### EPC-EXs^miR-17-5p^ were more effective than EPC-EXs in promoting viability and inhibiting apoptosis and necrosis of Hypoxia plus HG-treated ECs by activating the PI3K/Akt pathway

**Fig. 5:**
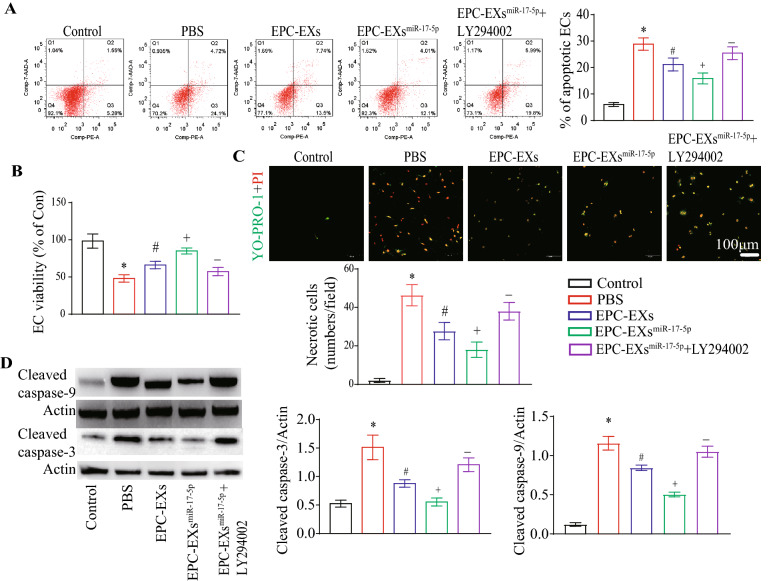
EPC-EXs^miR-17-5p^ increased viability, while reduced apoptosis of Hypoxia plus HG-injured ECs via activating PI3K/Akt pathway. **A** Representative images and summary data of apoptosis in ECs. **B** The viability of ECs. **C** Representative images and summary data of necrosis in ECs (YO-PRO-1, green; PI, red), Scale bar: 100 μm. **D** The levels of Cleaved caspase-3 and Cleaved caspase-9 in ECs after incubated with EPC-EXs, EPC-EXs^miR-17-5p^, and EPC-EXs^miR-17-5p^ + LY294002. ^*^p < 0.05 compared with the Sham group; ^#^p < 0.05 compared with PBS group; ^+^p < 0.05 compared with EPC-EXs group; ^−^p < 0.05 compared with EPC-EXs.^miR-17-5p^ group (**A**–**D** Two-way ANOVA, followed by Tukey’s post hoc test. Data represent the mean ± SEM, n = 3/group)

As shown in Fig. 5B, EPC-EXs^miR-17-5p^ increased the viability of Hypoxia plus HG-treated ECs more effectively than EPC-EXs (vs. EPC-EXs, Fig. 5B, p < 0.05), which was partially abolished by LY294002 (vs. EPC-EXs^miR-17-5p^, Fig. 5B, p < 0.05). To determine whether EPC-EXs^miR-17-5p^ incubation could inhibit the apoptosis of ECs under Hypoxia plus HG conditions, we analyzed the apoptotic rate and cleaved caspase-3 expression in ECs. We found that EPC-EXs could decrease the apoptotic rate and cleaved caspase-3 expression, which were compromised by Hypoxia plus HG treatment in ECs (vs. vehicle, Fig. 5A,  D, p < 0.05), and EPC-EXs^miR-17-5p^ displayed better efficacy (vs. EPC-EXs, Fig. 5E). A, 5D, p < 0.05). Moreover, pretreatment with LY294002 partially abolished these beneficial effects of EPC-EXs^miR-17-5p^ (vs. EPC-EXs^miR-17-5p^, Fig. 5A, D, p < 0.05). Further study showed that EPC-EXs could decrease the necrosis of ECs that had been compromised by Hypoxia plus HG treatment in ECs (vs. vehicle, Fig. 5C, p < 0.05), and EPC-EXs^miR-17-5p^ displayed better efficacy (vs. EPC-EXs, Fig. 5C, p < 0.05). Moreover, pretreatment with LY294002 partially abolished these beneficial effects of EPC-EXs^miR-17-5p^ (vs. EPC-EXsmiR-17-5p, Fig. 5C, p < 0.05).

Overall, these data indicate that EPC-EXs^miR-17-5p^ can promote the viability and inhibit the apoptosis and necrosis of Hypoxia plus HG-injured ECs by activating the PI3K/Akt signaling pathway.

### EPC-EXs^miR-17-5p^ were more effective than EPC-EXs in increasing the tube formation and migration of Hypoxia plus HG-treated ECs by activating the PI3K/Akt pathway

**Fig. 6:**
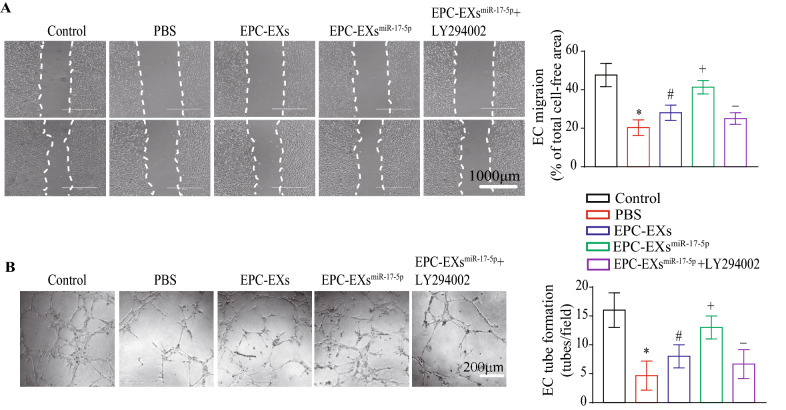
EPC-EXs^miR-17-5p^ increased migration and tube formation of Hypoxia plus HG-injured ECs via activating PI3K/Akt pathway. **A** Representative images and summary data of migration in ECs. Scale bar: 1000 μm. **B** Representative images and summary data of tube formation in ECs. ^*^p < 0.05 compared with the Sham group; ^#^p < 0.05 compared with PBS group; ^+^p < 0.05 compared with EPC-EXs group; ^−^p < 0.05 compared with EPC-EXs.^miR-17-5p^ group (**A**, **B** Two-way ANOVA, followed by Tukey’s post hoc test. Data represent the mean ± SEM, n = 3/group)

EC function analysis showed that EPC-EX incubation was able to increase EC migration, which was compromised by Hypoxia plus HG treatment (vs. vehicle, Fig. 6A, p < 0.05), and EPC-EXs^miR-17-5p^ displayed better efficacy (vs. EPC-EXs, Fig. 6A, p < 0.05). Moreover, pretreatment with LY294002 partially abolished these effects of EPC-EXs^miR-17-5p^ (vs. EPC-EXs^miR-17-5p^, Fig. 6A, p < 0.05). Additionally, we observed that EPC-EX incubation was able to increase EC tube formation, an effect that was compromised by Hypoxia plus HG treatment (vs. vehicle, Fig. 6B, p < 0.05), and EPC-EXs^miR-17-5p^ displayed better efficacy (vs. EPC-EXs, Fig. 6B, p < 0.05). Moreover, pretreatment with LY294002 partially abolished these effects of EPC-EXs^miR-17-5p^ (vs. EPC-EXs^miR-17-5p^, Fig. 6B, p < 0.05). These data indicate that EPC-EXs^miR-17-5p^ can promote the tube formation and migration of Hypoxia plus HG-injured ECs by activating the PI3K/Akt signaling pathway.

### EPC-EXs^miR-17-5p^ were more effective than EPC-EXs in promoting viability and inhibiting apoptosis of Hypoxia plus HG-treated C2C12 cells by activating the PI3K/Akt pathway

**Fig. 7:**
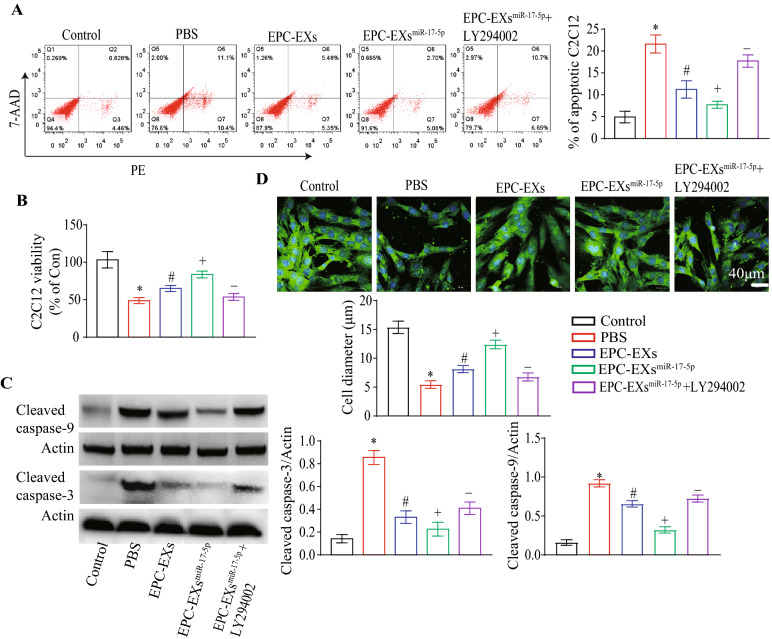
EPC-EXs^miR-17-5p^ increased viability, while reduced apoptosis of Hypoxia plus HG-injured C2C12 via activating PI3K/Akt pathway. **A** Representative images and summary data of apoptosis in C2C12. **B** The viability of C2C12. **C** The levels of Cleaved caspase-3 and Cleaved caspase-9 in ECs after incubated with EPC-EXs, EPC-EXs^miR-17-5p^, and EPC-EXs^miR-17-5p^ + LY294002. **D** The diameter was measured for C2C12 myotubes (MyHc, green) treated with EPC-EXs, EPC-EXs^miR-17-5p^, and EPC-EXs^miR-17-5p^ + LY294002. ^*^p < 0.05 compared with the Sham group; ^#^p < 0.05 compared with PBS group; ^+^p < 0.05 compared with EPC-EXs group; ^−^p < 0.05 compared with EPC-EXs.^miR-17-5p^ group (**A**–**D** Two-way ANOVA, followed by Tukey’s post hoc test. Data represent the mean ± SEM, n = 3/group)

As shown in Fig. 7A, EPC-EXs^miR-17-5p^ increased the viability of Hypoxia plus HG-treated C2C12 cells more effectively than EPC-EXs (vs. EPC-EXs, Fig. 7B, p < 0.05), an effect that was partially abolished by LY294002 (vs. EPC-EXs^miR-17-5p^, Fig. 7B, p < 0.05). To determine whether EPC-EXs^miR-17-5p^ could inhibit the apoptosis of C2C12 cells under Hypoxia plus HG conditions, we analyzed the cell apoptotic rate and cleaved caspase-9 and cleaved caspase-3 expression. We found that EPC-EXs were able to decrease the apoptotic rate and cleaved caspase-9 and cleaved caspase-3 expression of C2C12 cells that had been compromised by Hypoxia plus HG treatment (vs. vehicle, Fig. 7A, C, p < 0.05), and EPC-EXs^miR-17-5p^ displayed better efficacy (vs. EPC-EXs, Fig. 7A, C, D, p < 0.05). Moreover, pretreatment with LY294002 partially abolished these effects of EPC-EXs^miR-17-5p^ (vs. EPC-EXs^miR-17-5p^, Fig. 7A, C, p < 0.05). These data indicate that EPC-EXs^miR-17-5p^ can ameliorate the viability and inhibit the apoptosis of Hypoxia plus HG-injured C2C12 cells by activating the PI3K/Akt signaling pathway.

### EPC-EXs^miR-17-5p^ were more effective than EPC-EXs in elevating myotube formation of Hypoxia plus HG-treated C2C12 cells by activating the PI3K/Akt pathway

We further analyzed the myotube formation capability of C2C12 cells after EX incubation. As shown in Fig. 7D, we observed that EPC-EX incubation was able to increase the myotube size of C2C12 cells, an effect that was compromised by Hypoxia plus HG treatment (vs. vehicle, Fig. 7D, p < 0.05), and EPC-EXs^miR-17-5p^ displayed better efficacy (vs. EPC-EXs, Fig. 7D, p < 0.05). Moreover, pretreatment with LY294002 partially abolished the effects of EPC-EXs^miR-17-5p^ (vs. EPC-EXs^miR-17-5p^, Fig. 7D, p < 0.05). Taken together, these results suggest that EPC-EXs^miR-17-5p^ can increase the C2C12 myotube formation capability under Hypoxia plus HG conditions by activating the PI3K/Akt signaling pathway.

## Discussion

In this study, we found that miR-17-5p was downregulated in hind limb vessels and muscle tissue, and infusion of miR-17-5p-enriched EPC-EXs was more effective than EPC-EXs in improving blood perfusion, angiogenesis, and muscle regeneration in the hind limbs of DHI mice. In cultured HUVECs and C2C12 cells, miR-17-5p promoted the effects of EPC-EXs on protecting cells from Hypoxia plus HG-induced dysfunction and apoptosis by targeting SPRED1 and activating the downstream PI3K/Akt pathway.

In diabetic mellitus, hyperglycemia can exacerbate vascular dysfunction and apoptosis, which results in hind limb muscle tissue damage [32]. As the precursor cells of ECs, EPCs can mobilize into ischemia-injured blood vessels and improve the viability and tube formation of ECs through paracrine effects [33]. Of note, the activation and function of EPCs can be impaired by the pathological environments of various vascular diseases, such as ischemic stroke, diabetic mellitus [10], and hypertension [25]. EPCs from diabetic patients were shown to have low vascularization capability [34], and hyperglycemia can also induce the senescence of EPCs [35]. Recently, increasing evidence from us and others showed that EPC-derived exosomes (EPC-EXs) were capable of improving EC functions and promoting neovascularization in ischemic tissues [9, 36]. Moreover, the effects of exogenous EXs did not seem to be influenced by the pathological environment [37]. In the present study, we analyzed the role of EPC-EXs in diabetic hind limb ischemia (DHI)-induced blood vessel impairment. As the formation of tubular structures and blood vessels in ischemic tissue is closely associated with increased blood supplementation, we further evaluated the effects of EPC-EXs on DHI-induced hind limb blood flow reduction. In accordance with our expectations, we observed that EPC-EX administration could improve MVD, angiogenesis and blood perfusion while inhibiting EC apoptosis in the hind limb of the DHI mouse model. ECs are the main components of vessels and play a critical role in maintaining the function and integrity of the vascular unit, which has been shown to be particularly susceptible to hyperglycemia-induced damage [38]. In the present study, we built Hypoxia plus HG-injured EC models to mimic vessel damage after hind limb ischemia in diabetic mellitus [29]. We observed that Hypoxia plus HG-treated ECs exhibited decreased viability, migration and tube formation and increased apoptosis. EPC-EXs could inhibit the dysfunction and apoptosis of injured ECs, which was consistent with our in vivo study demonstrating that EPC-EX administration could improve DHI-induced hind limb vascular dysfunction. Evidence has demonstrated that the increased viability, migration and tube formation abilities and reduced apoptosis of ECs are highly associated with vascular integrity and neovascularization in ischemic damaged tissues [25]. Taken together, these data indicated that EPC-EXs could protect hind limb vessels from hyperglycemia- and hypoxia-induced injury, which added new evidence to the vascular protective effects of EPC-EXs [9].

The functional role of EPC-EXs in target cells relies on their cargos, such as RNAs and proteins. EPC-EXs are rich in cardioprotective and proangiogenic miRs, such as miR-126, miR-486, miR-210 and miR-133 [39]. EPC-EXs have been shown to protect ECs against hypoxia/reoxygenation-induced injury by transferring their carried miR-210 [40]. Recently, our group demonstrated that EPC-EXs exerted protective effects on hypoxia/reoxygenation-injured aging ECs through their carried ACE2 and miR-18a [26]. In a diabetic ischemic stroke mouse model, EPC-EXs could promote angiogenesis and neurogenesis in the peri-infarct area by transferring their carried miR-126 [9]. Taken together, these findings suggest that EPC-EXs may exert protective effects against hyperglycemia and ischemia-induced hind limb vascular and muscular injury by transferring their functional cargos, such as miR-126, miR-210, miR-486, and ACE2, a possibility that requires further investigation.

The functional role of EPC-EXs in target cells relies on their cargos (miRs, mRNAs, and proteins), and miRs are important functional cargos [9, 41]. MiR-17-5p was significantly reduced in the peripheral blood of diabetic patients [15] and has been shown to exert anti-inflammatory and anti-apoptotic effects in HG-treated human trophoblasts [16]. Additionally, a report demonstrated that miR-17-5p could ameliorate the viability and apoptosis of hypoxia/reoxygenation (H/R)-injured ECs [17]. In the present study, we observed that miR-17-5p was significantly decreased in hind limb vessels and muscle tissues of DHI mice, suggesting the potential role of miR-17-5p in protecting hind limb vessels and muscle tissues from hyperglycemia- and hypoxia-induced injury. Based on this evidence, we constructed miR-17-5p-enriched EPC-EXs and determined whether EPC-EXs^miR-17-5p^ could improve the protective effects of EPC-EXs on DHI-induced hind limb vascular impairment in vivo and on Hypoxia plus HG-induced EC dysfunction and apoptosis in vitro. We found that EPC-EXs^miR-17-5p^ were more effective than EPC-EXs in improving hind limb MVD, angiogenesis and blood perfusion while decreasing vascular EC apoptosis in a DHI mouse model and protecting ECs from Hypoxia plus HG-induced injury. These data highlight that engineered EPC-EXs with vascular protective miRs could enhance their vascular protective effects [9, 41].

Evidence has shown that diabetic patients are at high risk of hind limb muscle impairment and loss after DHI [42]. In addition to their protective effect against ischemia-induced vascular EC damage, EPC-EXs have been found to protect other cell types in ischemic tissue by regulating target cell functions. Recently, Halurkar MS. et al. reported that EPC-EXs could decrease Hypoxia plus HG-induced cytotoxicity and oxidative stress in astrocytes [43]. In a myocardial infarct rat model, EPC-EXs were found to promote the proliferation and angiogenesis of cardiac fibroblasts and exert therapeutic effects in myocardial infarction-induced cardiac injury [41]. Thus, we hypothesized that EPC-EXs may promote muscle regeneration after DHI by improving targeted muscle cell functions. In vivo, we analyzed the effects of EPC-EXs and EPC-EXs^miR-17-5p^ on DHI-induced hind limb muscle tissue injury. We found that EPC-EX administration could increase the size, weight, force production and structural integrity of ischemic muscle and reduce cell apoptosis after DHI. Our findings added new evidence to the protective effects of stem cell EXs on DHI-induced muscle damage [31]. Moreover, we also found that miR-17-5p enrichment could improve the protective effects of EPC-EXs on DHI-induced muscle tissue damage, which was in line with a previous study showing that miR-17-5p can promote skeletal muscle regeneration by increasing the differentiation of myoblast cells [18]. In addition, angiogenesis has been proven to play a pivotal role in muscle regeneration after DHI by providing oxygen and nutrients for the zone of ischemic injury [29]. Thus, the protective roles of EPC-EXs and EPC-EXs^miR-17-5p^ in DHI-induced muscle damage could also be associated with their beneficial effects on improving hind limb neovascularization and blood perfusion. It is well known that myoblast proliferation and differentiation into myotubes are key processes of muscle regeneration [44]. C2C12 myoblasts can proliferate and further differentiate into myocytes, form myotubes, and mature into myofibers [45, 46]. The myotube formation ability of C2C12 cells has been used to evaluate the repair and regeneration of muscle after injury [4]. Numerous studies have evaluated the regulation of muscle atrophy and damage by using differentiated C2C12 myotubes [47, 48], and C2C12 myotube diameter was used to assess the myotube formation ability of C2C12 cells [4]. Notably, C2C12 myoblast cells can differentiate into myotubes and have been used as an in vitro model of skeletal muscle differentiation [45, 49]. Thus, we carried out an experiment to measure the myotube formation ability of C2C12 cells by measuring the cell diameter. In the present study, we carried out experiments to determine the effects of EPC-EXs and EPC-EXs^miR-17-5p^ on regulating C2C12 cell viability, myotube formation ability, and apoptosis after Hypoxia plus HG treatment. We found that EPC-EX incubation markedly increased the viability and myotube formation abilities and reduced the apoptosis of Hypoxia plus HG-treated C2C12 cells. In addition, miR-17-5p enrichment could promote the protective effects of EPC-EXs on HG plus hypoxia-treated C2C12 cells, which is consistent with a previous study that demonstrated that miR-17-5p can elevate the differentiation of C2C12 myoblasts [18]. Taken together, our results indicated that EPC-EXs^miR-17-5p^ were more effective than EPC-EXs in promoting muscle regeneration and function in DHI mice by improving myoblast functions and blood perfusion while reducing myoblast cell apoptosis.

To further explore the underlying mechanism involved in the protective effects of EPC-EXs^miR-17-5p^ in Hypoxia plus HG-injured ECs and C2C12 cells, we carried out bioinformatics prediction analysis to identify the potential target gene of miR-17-5p. SPRED1 was found to be an overlapping target for miR-17-5p under the TargetScan, miRbase and miRanda databases. Based on the important roles of SPRED1 and its downstream PI3K/Akt signaling pathway in regulating EC proliferation, migration and tube formation abilities [24] and ischemic muscle cell repair [10], we further measured the SPRED1-PI3K/Akt signaling pathway in HG plus hypoxia-injured ECs and C2C12 cells after incubation with EPC-EXs^miR-17-5p^. In the present study, we clarified that miR-17-5p enhanced the effects of EPC-EXs on activating the phosphorylation of Akt in ECs and C2C12 cells injured by HG plus hypoxia by targeting SPRED1. Our findings demonstrate for the first time that EPC-EXs^miR-17-5p^ protect ECs and C2C12 cells from Hypoxia plus HG injury by targeting SPRED1 and activating the PI3K/Akt pathway, which is consistent with a previous study showing that the SPRED1-PI3K/Akt pathway is involved in regulating ECs and muscle cell functions [11, 24]. A PI3K inhibitor (LY294002) has been used to address the participation of the PI3K/Akt pathway in regulating multiple cell functions [50, 51]. Using a PI3K inhibitor, we further demonstrated that EPC-EXs enriched with miR-17-5p ameliorated HG plus hypoxia-induced EC (increasing viability, migration, and tube formation) and C2C12 cell (increasing viability and myotube size) dysfunction and apoptosis by activating the PI3K/Akt pathway. However, experiments involving the specific suppression of PI3K and the phosphorylation of Akt in ECs and C2C12 cells are needed to further confirm the pathway. We also found that EPC-EXs^miR-17-5p^ were more effective than EPC-EXs in decreasing cleaved caspase-3 expression in HG plus hypoxia-injured ECs and C2C12 cells, and a PI3K inhibitor diminished the effects of EPC-EXs^miR-17-5p^. It is well known that cleaved caspase-3 plays a key role in regulating cell apoptosis [52]. Thus, these data supported our in vivo findings that miR-17-5p promoted the effects of EPC-EXs in reducing DHI-induced hind limb cell apoptosis. Taken together, our findings added new evidence to the protective role of miR-17-5p in regulating EC [17] and C2C12 cell [18] functions and apoptosis. However, since the target proteins and downstream signaling pathways of miR-17-5p are complex under hyperglycemic and hypoxic conditions, our study on the mechanism of miR-17-5p-enriched EPC-EXs did not exclude other targets of miR-17-5p, such as inositol polyphosphate multikinase (IPMK)[53], polycystic kidney disease (PKD) [54], and phosphatase and tensin homolog (PTEN) [55], which may also be involved in regulating EC and C2C12 cell function. Thus, a dual-luciferase reporter assay and specific knockdown of SPRED1 in ECs and C2C12 cells are needed.

## Conclusion

Our findings in this study indicate that miR-17-5p-enriched EPC-EXs are more effective than EPC-EXs in ameliorating DHI mouse hind limb vascular and muscle injury by protecting hind limb vascular ECs and muscle cell functions and reducing cell apoptosis.

## Supplementary Information

**Additional file 1: Fig. S1**. Effects of EPC-EXs and EPC-EXs^miR-17-5p^ on hind limb vascular ECs proliferation and fast blood glucose of DHI mice. Scale bar: 30 μm. (A) Representative images and summary data of vascular EC proliferation in hind limb tissue of DHI mice. (B) The level of fast blood glucose. ^*^p < 0.05 compared with the Sham group; ^#^p < 0.05 compared with PBS group; ^+^p < 0.05 compared with EPC-EXs group;.^ns^ p ≥ 0.05 compared with the Sham group. (A, B Two-way ANOVA, followed by Tukey’s post hoc test. Data represent the mean ± SEM, n = 8 mice per group).

## Data Availability

All data generated or analyzed during this study are included in this published article.
