# Supplementary figures and images for: Enrichment of miR-17-5p enhances the protective effects of EPC-EXs on vascular and skeletal muscle injury in a diabetic hind limb ischemia model
Source: Biol Res. 2023 Apr 1;56:16. doi: 10.1186/s40659-023-00418-5 (PMC10067242; doi:10.1186/s40659-023-00418-5)

A

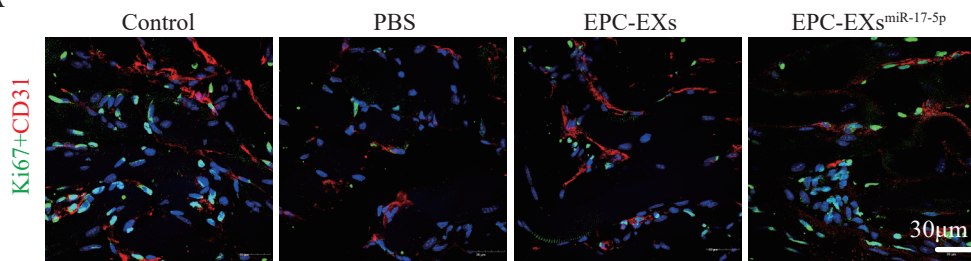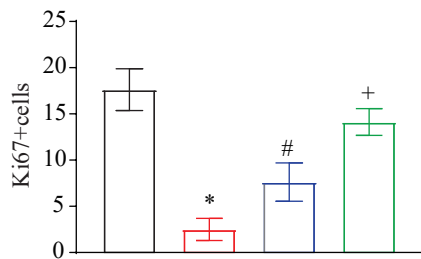

B

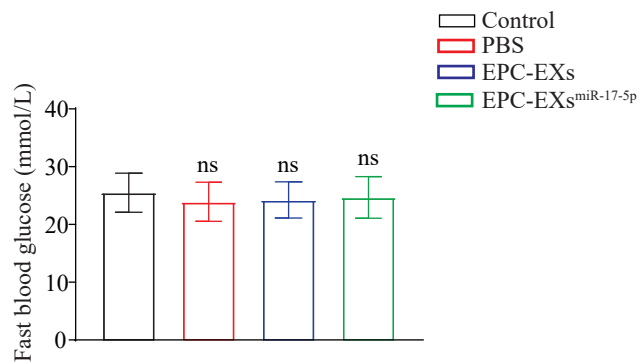

Supplement: Supplementary file 1 — Additional file 1: Fig. S1. Effects of EPC-EXs and EPC-EXsmiR-17-5p on hind limb vascular ECs proliferation and fast blood glucose of DHI mice. Scale bar: 30 μm. (A) Representative images and summary data of vascular EC proliferation in hind limb tissue of DHI mice. (B) The level of fast blood glucose. *p < 0.05 compared with the Sham group; #p < 0.05 compared with PBS group; +p < 0.05 compared with EPC-EXs group;.ns p ≥ 0.05 compared with the Sham group. (A, B Two-way ANOVA, followed by Tukey’s post hoc test. Data represent the mean ± SEM, n = 8 mice per group). [file 40659_2023_418_MOESM1_ESM.pdf]
